# Immunogenicity of the recombinant adenovirus fusion-expressing E0-E2 gene of the classical swine fever virus

**DOI:** 10.3389/fmicb.2022.1054651

**Published:** 2022-10-28

**Authors:** Heng Zhang, Dehua Yin, Huairui Qin, Ke Zhang, Zhaoyang Li, Guangchao Cui, Guangbin Ma, Peng Sun, Zhi Cao

**Affiliations:** ^1^Swine Disease R&D Center, Shandong SINDER Technology Co., Ltd., Qingdao, China; ^2^College of Animal Medicine, Qingdao Agricultural University, Qingdao, China; ^3^YEBIO Bioengineering Co., Ltd of Qingdao, Qingdao, China

**Keywords:** classical swine fever virus, E0-E2 gene, recombinant adenovirus vaccine, minimum immunization dose, immune duration

## Abstract

Adenovirus vector vaccines have been the mainstream research direction of CSF vaccines, due to the replication deficiency of adenovirus vectors, achieving double effects with the safety of inactivated vaccines and the efficacy of live vaccines. Therefore, the E0 and E2 genes were expressed by an adenovirus vector, a recombinant adenovirus E0-E2 (rAd-E0-E2) vaccine was constructed, and the minimum immunization dose and immune duration period were determined in this study. Forty healthy piglets were randomly divided into 8 groups (*n* = 5). Groups 1 ~ 5 were used to determine the minimum immunization dose, and 5 groups were inoculated with rAd-E0-E2 at different immune doses. Serum was collected at 7 d and 14 d after immunization to detect CSFV antibodies by ELISA, and piglets were challenged at 7 d post immunization. Groups 6 ~ 8 were immunized with 1 dose of rAd-E0-E2, the CSFV live attenuated vaccine C strain and saline to identify the immune duration period. Serum was collected at different time points after immunization, CSFV antibodies were detected by ELISA, and piglets were challenged at 8 months post immunization. Meanwhile, temperature, clinical symptoms and pathology were observed. The results of groups 1 ~ 5 showed that 1 piglet was protected after challenge, and 4 piglets exhibited high fever retention, typical CSFV symptoms and tissue lesions in the 1/50 dose group, whereas no clinical symptoms were observed in the 1/10 dose, 1/5 dose or 1 dose groups with 5/5 protection after challenge. The minimum dose was determined as 1/10 dose. The results of groups 6 ~ 8 showed that all piglets survived after challenge, but the antibody level of the rAd-E0-E2 strain was higher than that of the C strain at 8 months post immunization, and all piglets in the negative group developed the disease process after challenge. Overall, the minimum immunization dose of rAd-E0-E2 was 1/10 dose (3.16 × 10^6.0^ IFU) and the minimum immune dose was determined to be 1 dose (3.16 × 10^7.0^ IFU) to achieve the expected effects. The immune duration period of piglets immunized with 1 dose of rAd-E0-E2 was at least 8 months.

## Introduction

Classical swine fever (CSF) is a highly contagious, economically significant, multisystemic viral disease in swine, that is notifiable to the World Organization for Animal Health (WOAH; Blome et al., 2017; Ganges et al., 2020). The causative agent, CSF virus (CSFV), is an enveloped, positive-sense, single-stranded RNA virus. It belongs to the Pestivirus genus within the *Flaviviridae* family (Becher et al., 2003). At present, vaccination is still one of the effective ways to prevent CSF, but the symptoms of pigs infected by CSFV gradually trend towards chronic, insidious, and other atypical epidemic forms, thus making it imperative to develop a safe and effective vaccine to control the epidemic of CSF (Dong and Chen, 2007; Popescu et al., 2019).

At present, five structural proteins of CSFV have been identified, named N^pro^, C, E0 (E^rns^), E1 and E2. E0 and E2 are two main protective antigens for the viral-induced production of neutralizing antibodies in the organism, and are essential for virus adsorption and entry into host cells (Sun et al., 2013a). The host tropism of CSFV is related to E0, and the E0 protein can effectively neutralize E0 specific monoclonal antibodies and protect susceptible animals from infection (Xu et al., 2008). The E2 protein induces the production of neutralizing antibodies to the virus *in vitro*, and stimulates protective immunity against CSFV *in vivo*, protecting pigs from lethal CSFV infection (Huang et al., 2014; Henke et al., 2018; Tran et al., 2020). In addition, E0 and E2 chimeric antigens significantly improved the sensitivity of serological diagnosis and detected CSFV infection 7 days earlier than using E2 antigen alone (Lin et al., 2005; Sun et al., 2010, 2013a).

With the continuous development of molecular biology technology, more innovative vaccines for CSF have been developed and approved for use. The most widely used CSF vaccine in China is the live attenuated CSF vaccine (CSFV C strain, China), which is one of the most effective vaccines for CSF control. Moreover, with the cooperation of several companies, the CSFV E2 subunit vaccine (WH-09 strain) was developed using BEVS, whose production permit and new veterinary drug certificate were issued in 2020, and used in the swine industry. The inactivated CSFV E2 protein recombinant baculovirus vaccine (strain WH-09), jointly developed by Huazhong Agricultural University and Wuhan Keqian Biological Co., Ltd. and other companies, received a new veterinary drug certificate and production licence in 2020. Another subunit vaccine expressing CSFV E2 protein in HEK 293 T cells was developed by YEBIO Bioengineering Co., Ltd. of Qingdao, and is currently in the process of applying for a new veterinary drug certificate. In addition, human replication-deficient adenovirus type 5 live vector CSF vaccine and chimeric CSFV vaccine can induce complete immune protection. Adenovirus vector vaccines have been the mainstream research direction of CSF vaccines, due to the replication deficiency of adenovirus vectors, providing double effects with the safety of inactivated vaccines and the efficacy of live vaccines (Yan et al., 2020).

In our study, considering the deficiency of live vaccines and the high-safety of adenovirus vector vaccines, we examined a novel adenovirus live vector vaccine (rAd-E0-E2 strain) targeting to CSFV E0 and E2 genes. To verify the immune efficacy of the rAd-E0-E2 vaccine, the minimum immune dose and immune duration period were determined, with the hope of offering enough practical data for clinical application and provide a reference for subsequent application studies of this vaccine.

## Materials and methods

### Vaccines and viruses

A recombinant adenovirus vaccine (rAd-E0-E2 strain) expressing the E0-E2 gene of CSFV (live vector vaccine, 3.16 × 10^7.0^ IFU per dose) was lyophilized and preserved at the Swine Diseases R&D Center of SINDER. Live CSF vaccine (splenic lymphogen source, C strain), was produced by the lyophilized vaccine workshop of SINDER. CSFV Shimen strain (CVCC AV1411) blood virus (F115 generation, ≥10^5.0^ MLD), was purchased from the China Veterinary Culture Collection Center (CVCC).

### Animals

Six-to eight-week-old healthy and susceptible weaned piglets (*n* = 60), which were free of CSFV, porcine circovirus 2 (PCV2), and porcine reproduction and respiratory syndrome virus (PRRSV) infection. All piglet experiments were performed at YEBIO Bioengineering Co., Ltd. of Qingdao and in accordance with the protocols approved by the Animal Care and Ethics Committee of the China Animal Health and Epidemiology Center under number CNASL1005.

### Package and identification of recombinant adenovirus

The primers for the CSFV E0 and E2 genes were designed by Oligo 5.0 according to the CSFV genome sequence (Table 1) and synthesized by Sangon Biotech (Shanghai) after codons were optimized. The E0 and E2 protein genes were amplified by PCR and cloned sequentially into the adenovirus shuttle vector pAdTrack-CMV. The positive recombinant shuttle plasmid was named pAdTrack-E0-E2. pAdTrack-E0-E2 and the skeleton plasmid were cotransfected into HEK293 cells, and recombinant adenovirus was harvested when typical CPEs were observed, such as those that were rounded and broken. Then, the specificity of the recombinant adenovirus rAd-E0-E2 strain was identified by indirect immunofluorescence (IFA).

**Table 1:** Primers for CSFV E0 and E2 proteins.

Genes	Primer sequences (5′-3′)	Restriction sites
CSFV-E0	F:AAATCTAGAATGGAAAATATAACTCAAT R:CCCGGTACCGGCATAAGCGCCAAAC	*Kpn* I，*Bgl* II
CSFV-E2	F:AAAGGTACCAGGGGACAGATCGTGCAT R:CCCGCGGCCGCGGCGAGTTGTTCTGTTAG	*Not* I，*Bgl* II

### Determination of virus content

HEK293 cells were cultured in DMEM containing 10% NBS, and cells were seeded in 24-well plates and incubated at 37°C in 5% CO_2_ for 2 to 3 h. Then, 100 μl/well of 10-fold-diluted (from 10^−3^ to 10^−7^) rAd-E0-E2 strain virus in DMEM was added to HEK293 cells in 24-well plates for three replicates of each dilution, accompanied by cell-negative control wells. After incubation for 48 h, indirect immunofluorescence (IFA) was used to detect cell infection and calculate the viral content.

Virus content per well $=\frac{\mathrm{Numbers of positive cells in vision field}}{\;\mathrm{total number of fields of vision}}$ × $\frac{\;\mathrm{standard well area}}{\;\mathrm{visual field area}}$ × $\frac{\mathrm{dilution ratio}}{\;0.1\mathrm{mL}}$

Virus content = $\frac{\mathrm{sum}\;\mathrm{of virus content}\;\mathrm{per}\;\mathrm{well}\;}{\;\mathrm{number of wells}}$

### Piglet vaccination and challenge

Forty healthy piglets were randomly divided into 8 groups (*n* = 5), groups 1 ~ 5 were used to determine the minimum immunization dose and groups 6 ~ 8 were used to determine the immune duration period. The detailed experimental design is provided in Table 2.

**Table 2:** Experiments design of determination of minimum immune dose and immune duration period.

Experimental content	Groups	Immune dose	Viral titer(IFU/dose)	Number	Challente time
Minimum immune dose group	1	1/50 dose	6.32 × 10^5^	5	Day 7 post vaccination
	2	1/10 dose	3.16 × 10^6^	5	
	3	1/5 dose	6.32 × 10^6^	5	
	4	1 dose	3.16 × 10^7^	5	
	5	Saline	0	5	
Immune duration group	6	rAd-E0-E2 strain	3.16 × 10^7^	5	8th month post vaccination
	7	C strain	1 dose	5	
	8	Saline	0	5	

### Detection of antibodies in serum samples

Blood was collected from the precaval vein of piglets at 7 d and 14 d post immunization in groups 1 ~ 5 and at 7 d, 14 d, 21 d, 30 d, 60 d, 90 d, 120 d, 150 d, 180 d, 210 d and 240 d post immunization in groups 6 ~ 8, and the serum was separated. CSFV-specific antibodies were tested with a classical swine fever virus antibody test kit (IDEXX, United States) following the manufacturer’s instructions.

### Examination of clinical signs and pathological changes

The temperatures of all experimental animals were measured from 2 days before the challenge to 16 days after the challenge. The spirit, food and water intake, faeces, and morbidity of pigs were observed daily, and all surviving pigs were dissected at 16 days post challenge. The tissue lesions were observed, and the survival rate was recorded.

### Histopathology

The representative organs were collected and fixed in 10% neutral formalin at room temperature for 24 h. The fixed tissues were embedded in paraffin wax, stained with haematoxylin and eosin (H&E), and examined by light microscopy.

### Statistical analysis

Data are presented as the mean ± standard deviation (SD) of five replicates using Origin software.

## Results

### Identification and loads of recombinant adenovirus rAd-E0-E2 strain

**Figure 1:**
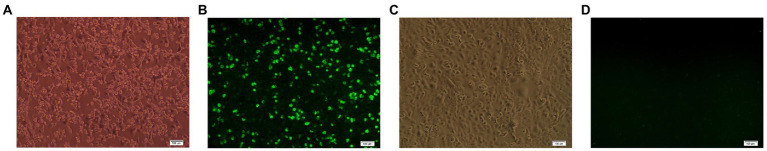
IFA identified with the recombinant adenovirus rAd-E0-E2. **(A)** Cytopathic effects (CPE) in HEK 293 cells transfected with pAdEasy-E0-E2. **(B)** Green fluorescence in HEK 293 cells transfected with pAdEasy-E0-E2. **(C)** No CPE in normal cell controls. **(D)** No fluorescence in normal control cells. Scale bar: 100 μm.

After pAdEasy-E0-E2 and pAdTrack-CMV were transfected into HEK293 cells by lipidosomes, obvious CPE was found in HEK 293 cells on the 10th day post transfection (Figure 1A), and green fluorescence was observed in the cells transfected with pAdEasy-E0-E2 (Figure 1B), while there was no CPE or fluorescence in the control cells without plasmid transfection (Figures 1C,D). The results showed that the recombinant adenovirus rAd-E0-E2 strain was obtained. HEK293 cells were inoculated with10^5^-diluted adenovirus, and the loads of recombinant adenovirus rAd-E0-E2 was 10^8.5^ IFU/ml.

### Determination of the minimum immune dose of the rAd-E0-E2 live vector vaccine

#### Detection of specific antibodies

**Figure 2:**
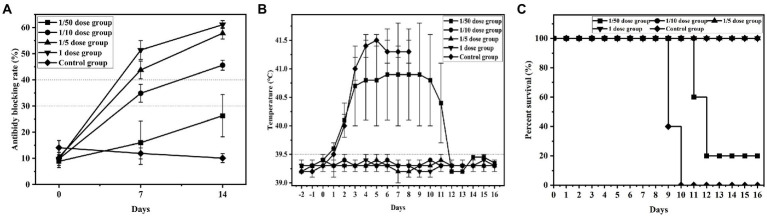
Detection results of minimum immune dose groups. Piglets were immunized with different doses of recombinant adenovirus live vector vaccine (1/20 dose, 1/10 dose, 1/2 dose, 1 dose), and control group were immunized with saline. All groups was challenged at 14 dpi post immunization. **(A)** Variation in the average blocking rate of antibodies in serum was measured at 7 dpi and 14 dpi. **(B)** Rectal temperature was measured during the 16-day observation period, and **(C)** the survival rate was recorded. Data are presented as the mean ± standard deviation (SD).

Groups 1 ~ 5 were used to determine the minimum immune dose (1/50 dose, 1/10 dose, 1/5 dose, 1 dose and control group) and serum was collected to detect CSFV-specific antibodies by ELSA. The Results showed that the antibody-positive rate of group 1 (1/50 dose) was 1/5 with a 26.3% antibody blocking rate, and the specific antibody of all piglets was positive in groups 2 ~ 4 (1/10 dose, 1/5 dose and 1 dose) with 45.6, 57.8 and 61.1% blocking rates, respectively. However, 0 piglets were positive in group 5 (control group), with a ≤ 12.0% blocking rate. These results indicated that specific antibodies of piglets vaccinated with 1/10 dose of live vector vaccine (rAd-E0-E2) were close to positive, and ≥ 1/5 dose were positive at 7 d post vaccination (Figure 2A).

#### Temperature monitoring

Compared to the normal temperature of 39.5°C, group 5 (control group) maintained a higher fever until death, and 4 piglets in group 1 (1/50 dose) had a higher fever than 40°C at 2 d post challenge and maintained a high fever until 16 d post challenge, exhibiting continued fever. Conversely, the temperatures of group 2 ~ 4 (1/10 dose, 1/5 dose, and 1 dose) piglets were normal, which indicated that the 1/10 dose of live vector vaccine (rAd-E0-E2) could protect piglets from CSFV infection (Figure 2B).

#### Mortality examination

Four pigs in group 1 died at 11 d ~ 12 d post challenge, but no deaths occurred in groups 2, 3, and 4. All piglets in group 5 died at 9 ~ 10d post challenge, showing typical CSF symptoms, which demonstrated that piglets immunized with 1/10 dose or above of the rAd-E0-E2 strain for 7 d were completely protected from lethal CSFV attack (Figure 2C).

### Determination of the immune duration of the rAd-E0-E2 live vector vaccine

#### Detection of specific antibodies

**Figure 3:**
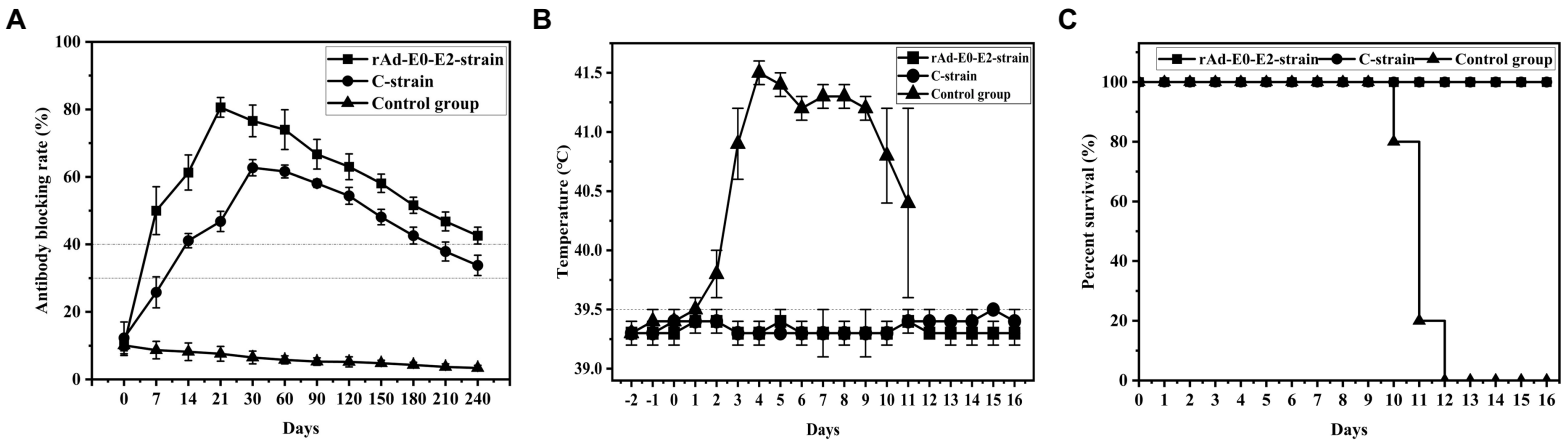
Determination of the immune duration of the rAd-E0-E2 live vector vaccine. Piglets were immunized with different types of live vector vaccines (rAd-E0-E2 strain and C strain), and the control group was immunized with saline. All groups were challenged at 8 months post immunization. **(A)** Variation in the average blocking rate of antibodies in serum was measured at 7 dpi and 14 dpi, in which a higher antibody-blocking rate indicated higher antibodies level. **(B)** Rectal temperature was measured during the 8-month observation period, and **(C)** the survival rate was recorded. Data are presented as the mean ± standard deviation (SD).

Groups 6 ~ 8 were vaccinated with live vector vaccine (rAd-E0-E2, 1 dose), live vector vaccine (CSFV C strain, 1 dose) and saline, and were used to determine the immune duration period. The results showed that CSFV-specific antibodies of piglets were positive at 7 d post vaccination and sustained at least 8 months in group 6. The antibody-positive rate of group 7 was 4/5 at 7 d post vaccination and subsequently decreased to suspicious, while the antibody rate of 5/5 piglets was positive. However, the CSFV-specific antibody of group 8 was negative throughout the experiment (Figure 3A). These results showed that CSFV-specific antibody of piglets vaccinated with 1 dose of live vector vaccine (rAd-E0-E2) could be sustained for at least 8 months, which was superior to that of piglets vaccinated with live vaccine (CSFV C strain, 1 dose).

#### Temperature monitoring

In the immune duration experiment, the temperatures of groups 6 and 7 were normal while group 8 had a fever higher than 40°C and exhibited continued fever, suggesting that piglets vaccinated with the live vector vaccines rAd-E0-E2 strain and C strain did not exhibit typical fever symptoms (Figure 3B).

#### Mortality examination

Groups 6 and 7 had no CSF clinical symptoms or pathological changes post challenge, and the survival rate was 100%. All piglets in group 8 died at 10 ~ 12 d post challenge, showing typical CSF symptoms such as continued high fever. The results showed that both the rAd-E0-E2 strain and C strain could also protect immunized pigs from CSFV for at least 8 months (Figure 3C).

### Clinical symptoms post challenge

**Figure 4:**
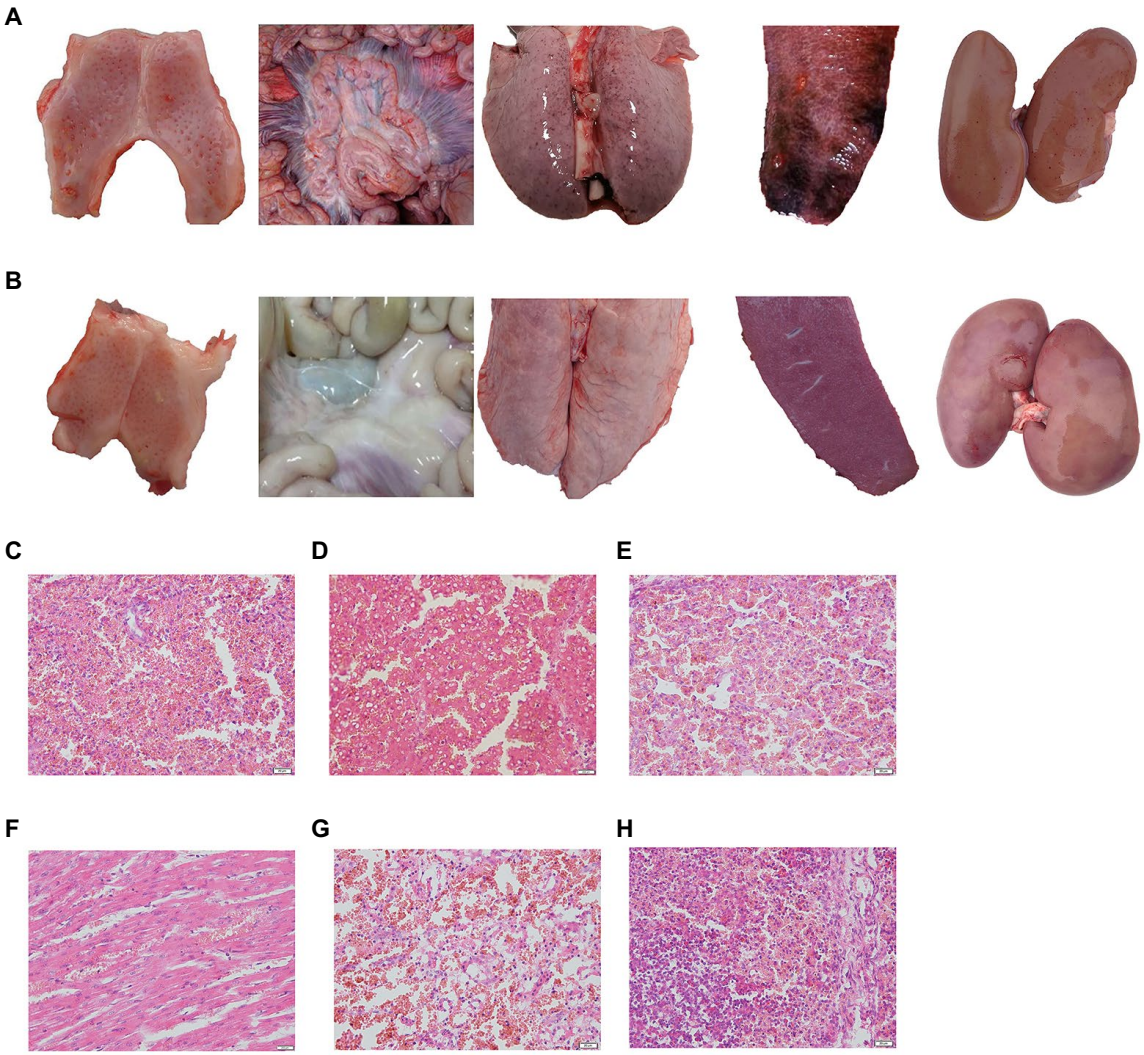
Typical pathological lesions of tonsil, intestinal lymph node, lung, spleen, and kidney in control group **(A)** and normal piglets **(B)**, and histopathological examination of the spleen **(C)**, liver **(D)**, lung **(E)**, heart **(F)**, kidney **(G)**, and lymph gland **(H)**. Scale bar: 200 μm.

The challenged piglets of group 5 (5/5) and group 1 (4/5) showed CSF-related clinical symptoms including high body temperature, depression, conjunctivitis, diarrhoea and loss of appetite, and obvious pathological changes, such as marginal spleen infarction and haemorrhagia in the kidney and lymph gland (Figure 4A), which were observed in groups 5 and group 8. However, no clinical symptoms or pathological changes were observed in groups 2, 3 and 4, which suggested that a 1/10 dose of live vector vaccine (rAd-E0-E2) had a protective effect on pigs (Figure 4B).

No CSF-related symptoms or pathological changes in tissues were observed in groups 6 and 7 at 8th month post immunization, while all piglets exhibited high fever at 40°C from 2 ~ 3 days post challenge, and symptoms of CSF appeared in group 8 (Figures 4C–H). These data suggested that piglets vaccinated with the live vector vaccines rAd-E0-E2 and C strain can protect piglets from CSFV attack for at least 8 months.

## Discussion

CSFV E0 protein is the only glycoprotein secreted into the culture supernatant of CSFV-infected cells. It can stimulate pigs to produce neutralizing antibodies against CSFV infection (Xu et al., 2020; Reuscher et al., 2021). The glycoprotein E2 has been proven to be the main structural protein of CSFV and can induce a protective response in pigs (Panyasing et al., 2018). As a research hotspot in recent years, the recombinant live vector recombinant vaccine of CSF is a novel vaccine prepared by recombining the virus antigen gene into a live vector that can express exogenous virus protein, which can stimulate the body to produce a specific immune response (Wei et al., 2021). At present, the structure and function of the adenovirus genome have been studied thoroughly; meanwhile, human type 5 nonreplicating adenovirus vectors have attracted researchers’ attention because of their safety, wide host range and stability (Zhu et al., 2017; Yan et al., 2020). Previous studies have shown that adenovirus vectors can induce strong specific cellular and humoral immune responses, which are widely used in the construction of new vaccines against severe zoonotic diseases (Gabitzsch et al., 2011; Lapuente et al., 2018; Zhang et al., 2018).

In our study, the E0 and E2 genes were inserted into the human type 5 adenovirus vector, the complete virions were packaged into HEK 293 cells (Figure 1A), and high-titre adenoviruses were harvested. After 10 continuous passages, we constructed a recombinant adenovirus vaccine based on CSFV E0 and E2 proteins using an adenovirus vector (rAd-E0-E2), and conducted detailed experiments to determine the minimum immune dose and immune duration period in piglets. Overall, the recombinant adenovirus vector vaccine (rAd-E0-E2) showed no difference from the CSF splenic lymphoid vaccine (CSFV C strain) in protecting piglets.

To determine the minimum immune dose of the rAd-E0-E2 live vaccine, we designed four doses, including a 1/50 dose, 1/10 dose, 1/5 dose and 1 dose, and sera were collected at 7 dpi and 14 dpi. The CSFV-specific antibody was positive partly from 7 days post immunization, and all piglets were CSFV antibody-positive at 14 dpi with a blocking rate ≥ 40% (Figure 2A). Although piglets immunized with the 1/10 dose of live vector vaccine (rAd-E0-E2 strain) were not CSFV antibody-positive completely, the piglets were protected completely from CSFV lethal attack without high-temperature symptoms (Figures 2B,C), which indicated that cellular immunity or mucosal immunity was enhanced, covering the shortage of lower humoral immunity. Therefore, a 1/10 dose of live vector vaccine can offer protection in a short time, reducing the probability of CSFV infection during the immune window period. On the other hand, the immune duration period of the rAd-E0-E2 vaccine lasted at least 8 months, which was comparable to that of the live vector vaccine C-strain, and all piglets survived without any CSF-related symptoms (Figures 3B,C). In terms of CSFV-specific antibodies, a higher blocking rate was observed in the rAd-E0-E2-immunized serum than in the serum-immunized live attenuated vaccine C-strain (Figure 3A).

Sun et al. constructed a recombinant adenovirus vector vaccine expressing E2 protein (rAdV-SFV-E2) and evaluated the vaccine performance in rabbits and pigs (Sun et al., 2013b). In a rabbit vaccination-challenge model, one dose of 10^6^ TCID_50_ rAdV-SFV-E2 induced early (as early as 9 days) and long-lasting (up to 189 days) immune responses, and the minimum dose was determined to be one dose of 2.5 × 10^5^ TCID_50_. Moreover, pigs were conferred immunity with two doses of 10^7^ TCID_50_. However, the rAd-E0-E2 live vector vaccine in our study elicited robust immune responses as early as 7 days, and the immune duration period lasted up to 8 months, which suggested that the rAd-E0-E2 vaccine can offer comprehensive protection compared to a recombinant adenovirus vector vaccine expressing a single protein. In addition, a long immune period can be achieved, which provides convenience for large-scale herds and sharply reduces costs.

In conclusion, a recombinant adenovirus live vector vaccine (rAd-E0-E2 strain) was successfully constructed. The minimum immunization dose of the rAd-E0-E2 vaccine was 1/10 dose (3.16 × 10^6.0^ IFU) to protect piglets from lethal CSFV challenge, and the duration of immunization in piglets immunized with 1 dose of rAd-E0-E2 was at least 8 months. These data provide scientific material for the clinical application of the vaccine.

## Data availability statement

The raw data supporting the conclusions of this article will be made available by the authors, without undue reservation.

## Ethics statement

The animal study was reviewed and approved by Animal Care and Ethics Committee of the China Animal Health and Epidemiology Center, under the number CNASL1005.

## Author contributions

ZC, HQ, GM, and DY: data curation. HQ, KZ, and ZL: formal analysis. HZ, GC, and PS: methodology. ZC, ZL, and GC: project administration. ZC: supervision. HZ, DY, HQ, and KZ: writing–original draft. HZ and ZC: writing–review and editing. All authors contributed to the article and approved the submitted version.

## Funding

This work was supported by the Post Expert of Disease Control in Shandong Technique System for Pig Industry (Project No. SDAIT-08-07) and the National Natural Science Foundation of China (32002269).

## Conflict of interest

HZ, ZL, GC, and GM were employed by the company Shandong SINDER Technology Co., Ltd., and PS was employed by the company YEBIO Bioengineering Co., Ltd of Qingdao.

The remaining authors declare that the research was conducted in the absence of any commercial or financial relationships that could be construed as a potential conflict of interest.

## Publisher’s note

All claims expressed in this article are solely those of the authors and do not necessarily represent those of their affiliated organizations, or those of the publisher, the editors and the reviewers. Any product that may be evaluated in this article, or claim that may be made by its manufacturer, is not guaranteed or endorsed by the publisher.
